# Population balance modelling captures host cell protein dynamics in CHO cell cultures

**DOI:** 10.1371/journal.pone.0265886

**Published:** 2022-03-23

**Authors:** Sakhr Alhuthali, Cleo Kontoravdi

**Affiliations:** Department of Chemical Engineering, Imperial College London, London, United Kingdom; University College Dublin, IRELAND

## Abstract

Monoclonal antibodies (mAbs) have been extensively studied for their wide therapeutic and research applications. Increases in mAb titre has been achieved mainly by cell culture media/feed improvement and cell line engineering to increase cell density and specific mAb productivity. However, this improvement has shifted the bottleneck to downstream purification steps. The higher accumulation of the main cell-derived impurities, host cell proteins (HCPs), in the supernatant can negatively affect product integrity and immunogenicity in addition to increasing the cost of capture and polishing steps. Mathematical modelling of bioprocess dynamics is a valuable tool to improve industrial production at fast rate and low cost. Herein, a single stage volume-based population balance model (PBM) has been built to capture Chinese hamster ovary (CHO) cell behaviour in fed-batch bioreactors. Using cell volume as the internal variable, the model captures the dynamics of mAb and HCP accumulation extracellularly under physiological and mild hypothermic culture conditions. Model-based analysis and orthogonal measurements of lactate dehydrogenase activity and double-stranded DNA concentration in the supernatant show that a significant proportion of HCPs found in the extracellular matrix is secreted by viable cells. The PBM then served as a platform for generating operating strategies that optimise antibody titre and increase cost-efficiency while minimising impurity levels.

## 1 Introduction

Monoclonal antibodies (mAbs) and derived products such as antibody-drug conjugates, Fc-fusion proteins and antibody fragments, are widely used in many diagnostic and therapeutic applications [1]. Chinese hamster ovary (CHO) cells are the most commonly used host for industrial mAb production, accounting for 84% of currently approved products [2]. mAb yield increased from 50 mg/L in 1986 to 3–10 g/L in 2010s as a result of a variety of genetic and process optimisation techniques that led to increased cell density and specific mAb productivity [3–5]. However, achieving higher cell densities has also led to the accumulation of cell-derived impurities over culture duration, posing challenges for downstream separation [6,7].

Host cell proteins (HCPs) are a heterogeneous mixture of proteins secreted by viable cells and intracellular proteins released from dead and lysed cells toward the end of the culture [7]. In their majority, HCPs are removed in the downstream step of protein A affinity chromatography, however, some persist after this unit operation and their removal requires further chromatographic separation steps [8]. Certain HCPs have proteolytic activity and can cause product fragmentation (and subsequent aggregation) while others can lead to immunogenic responses in patients if not fully removed [6]. The concentration and properties of residual HCPs present in process intermediates and the drug product are therefore of significant interest [9,10].

HCP concentration typically increases with cell culture duration and correlates with the number of dead cells, even though HCPs are also known to be secreted by viable cells [6,11]. Although the goal of upstream operations has typically been to increase yield, which often involves improved feeding regimes to increase culture longevity, this is not necessarily beneficial from the point of view of downstream processing effort and cost. The ability to quantify the trade-off between increased yield and raised purification effort could therefore support decision-making during process development, such as optimal harvest time. Including the level of impurities in upstream process models paves the way for multi-objective optimisation of both upstream processing steps and the entire flowsheet to reduce the process development and manufacturing cost of therapeutic proteins and increase overall process efficiency.

To this end, modelling of cell culture dynamics has been a valuable tool in enhancing our understanding of cellular responses to process changes [12,13]. We propose that modelling can be used to quantify this trade-off between gains in upstream operations and burden on downstream purification by predicting the accumulation of dead cells, which is responsible for HCP release extracellularly and is often underestimated by trypan blue dye exclusion methods [14]. However, such a model would also need to account for HCP secretion by viable cells and, by extension, accurately represent cell volume, which is correlated to the total protein content per cell [15].

Population balance modelling (PBM) has been developed to model different complex dynamic systems that are characterised by distributions [16–22]. These models have a time-dependent probability density function, which represents a state variable such as cell volume or DNA content. The distribution changes with time as a result of growth, death and birth mechanisms [18,23,24]. PBM has been used extensively to describe cell population dynamics in a variety of bioreactor systems and across different microorganisms [25–28]. However, the application of PBM with a single internal variable model is limited to balanced growth conditions [29,30], under which it is reasonable to assume that cell division follows a fixed normal distribution of cell size [31]. This is not applicable to the fed-batch culture of CHO cells, in which cell volume increases significantly in late-stage culture due to an increase in culture osmolality [32–34]. Mantzaris et al. and Zhu et al. were among the first to model the evolution of cell volume distribution in yeast, which led to the model being split into two different stages, smaller cells with high proliferation rate and larger ones with high recombinant protein productivity [28,30].

These papers along with others [29,35,36] emphasise the importance of uncoupling cell growth in size from cell density when growth medium nutrient and metabolite concentration change. This modelling approach is called multi-stage PBM, in which the number of model equations increases with the number of population balance stages considered. Given previous observations on the link between cell volume, growth rate and culture osmolality [34,37–39], we propose a simpler approach for modelling the effect of osmolality. Specifically, we propose the inclusion of culture osmolality in the growth rate equation and division function to capture (a) growth rate reduction and (b) cell volume increase under hyperosmolar culture conditions.

We have previously shown that CHO cells divide at a lower rate and larger cell volume as extracellular osmolality increases (i.e., 400 mOsm kg ^-1^) before cell division ceases at higher osmolality values (~500 mOsm kg ^-1^) [34]. This cell volume increase is observed with every new generation of cells and can be considered in the division function of the PBM by adjusting the mean volume and standard deviation at which cells divide. These values are typically taken from literature, but can be updated based on experimental data on cell volume distribution for the cell line and process conditions at hand [36,40]. It is also possible for the division mean and standard deviation to be formulated as a function of culture osmolality such that an increase in CHO cell volume leads to division only if extracellular conditions (i.e., substrate and metabolite concentrations) are conducive to cell proliferation. Hence, we present a single-stage volume-based population balance model formulation for mAb-producing CHO cell cultures. The PBM captures cell density and volume growth dynamics, mAb production, as well as the accumulation of HCPs extracellularly as a measure of downstream burden under two culture temperatures: 36.5°C and 32°C. The model is then used to understand the mechanisms by which HCPs accumulate in the extracellular matrix and to compare optimal culture conditions for different objectives. We anticipate that the model can guide decision-making with respect to optimal harvest time to maintain high product recovery and HCP impurity clearance and, with further data on HCP stability, can refine our understanding of their origin.

## 2 Materials and methods

### 2.1 Model formulation

The main equation of PBM for a single compartment and a single internal variable, cell volume in this case, is:

Accumulation+Growth+Division+Death+Dilution=Birth (1)

The mathematical representation of these terms is given by Eq (2).

$$\frac{\partial n(v,t)}{\partial t}+\frac{\partial }{\partial v}(g(v,s)n(v,t))+\Gamma (v,s)n(v,t)+D(s)n(v,t)+\frac{F_{in}}{V}n(v,t)=2\int_{v_{min}}^{v_{max}}P(v,v^{\prime })\Gamma (v^{\prime },s)n(v^{\prime },t)dv^{\prime }$$ (2)

*n*(*v*,*t*) is number of cells in million per unit volume of the continuous internal normalised phase (normalised cell volume) per unit volume of the discrete phase (L of culture) at time t. The integral of *n*(*v*,*t*) is called the 0^th^ moment, which gives the number density of cells across all cell sizes [41]. The terms *v*′ and *v* represent the volume of mother and daughter cells, respectively, with further details provided in section 2.1.3. The integration of cell volume in the model is important because metabolic flux across the membrane, biosynthetic capacity and nutrient exchange depend on cell size [42,43]. The nucleus, mitochondria, endoplasmic reticulum and gene expression scale with cell size in human and yeast cells [44–48].

#### 2.1.1 Growth rate

Most of cell growth rate equations used in PBM [49–53] involve a distributed linear domain like the volume shown in Eq (3) below. The linear cell volume growth function for CHO cells was suggested by Anderson et al. similarly to studies in other microorganisms [54,55].


$$g(v,s)={\mu_{max}f}_{lim}f_{inh}v$$ (3)

$$f_{lim}=\frac{C_{asn}}{C_{asn}+K_{asn}}$$ (4)

$$f_{inh}=\frac{K_{amm}}{{C_{amm}+K}_{amm}}\left(\frac{-Osm}{K_{Osm}}+2.2\right)\;315\mathrm{mOsm}\;{\mathrm{kg}}^{‐1}<\mathrm{Osm}<560\;\mathrm{mOsm}\;{\mathrm{kg}}^{‐1}$$ (5)

The population growth rate *g*(*v*,*s*) is in units of *h*^−1^ due to the volume domain being normalised. The growth rate depends on substrate limitation (Eq (4)), metabolite inhibition (Eq (5)) and the normalised cell volume *v*. Asparagine is the main growth-limiting substrate and ammonia inhibits growth for this cell line (see Kyriakopoulos and Kontoravdi [56]). The *K*_*asn*_ and *K*_*amm*_ are the Monod and inhibition constants (*mM*), respectively. Lopez-Meza et al. linked growth to substrate concentration for wild-type and protein-producing CHO cells whereas Stamatakis et al. qualitative PBM assumed cell growth as a function of volume alone [57,58]. In Eq (5) for the growth inhibition function, *f*_*inh*_, the effect of osmolality is accounted for in last term in accordance with literature and is further discussed in the osmolality section [34,38]. The constant *K*_*Osm*_ in Eq (5) has a value of 256 mOsm kg ^-1^. Eq (5) is only valid for the given osmolality range. This is because our cell culture medium has initial osmolality value of 320 mOsm kg^-1^ and osmolality above 500 will lead to inhibitory condition, *f*_*inh*_ turns to zero at 565 which is well above the operating range. It is worth mentioning that the parameters involved in Eq (5) should be experimentally verified before implementation for different cell line or culture medium.

Linardos et al. reported the exponential relationship between the cell death rate and average cell age [59,60]. The death rate is an exponential function as shown in Eq (6) and positively correlated to the reduction in proliferation rate [59,61,62]. Eq (7) accounts for cell lysis rate of dead cells. The accumulation of lysed cells can be quantified by Eq (8).


$$D(s)=k_{d\;max}\;e^{\left(\frac{-\mu_{max}f_{lim}f_{inh}}{k_{d}}\right)}$$ (6)

$$\frac{dN_{d}}{dt}=\int_{v_{min}}^{v_{max}}D(s)n(v,t)dv-k_{l}\;N_{d}$$ (7)

$$\frac{{dN}_{l}}{dt}=k_{l}\;N_{d}$$ (8)

In Eq (6), *k*_*d max*_ (*h*^−1^) is the maximum death rate and *k*_*d*_ (*h*^−1^) is another constant specific to the cell line which is the growth rate at which the death rate is at a maximum value. *k*_*l*_ (*h*^−1^) in the Eq (7) is the cell lysis rate. *N*_*d*_ (10^6^ *cell L*^−1^) is the density of dead cells given in the same unit as viable cell density *N* in Eq (9). The cell density (10^6^ *cell L*^−1^) can be found by integrating the distributions of viable cell population from the minimum to the maximum cell volume as shown in Eq (9). In this case, the minimum volume is zero and the maximum is unity. The boundary condition in Eq (10) signifies that there is not a cell which has volume of zero at any time.


$$N=\int_{v_{min}}^{v_{max}}n(v,t)dv$$ (9)

$$n_{a}(0,t)=0$$ (10)

The following two equations are the initial conditions for dead and lysed cells. Eq (13) is used to calculate the average cell volume.


$$N_{d}(v,0)=0$$ (11)

$$N_{l}(v,0)=0$$ (12)

$$\bar{v}=\frac{\int_{v_{min}}^{v_{max}}v\;n(v,t)dv}{N}$$ (13)

$\bar{v}$ is the average cell volume which is normalised and the corresponding value in *μm*^3^ can be obtained by multiplying the value by the maximum cell volume 6,044 *μm*^3^, based on 22.6 μm maximum diameter. This maximum diameter is well above typically reported values to ensure that the entire cell population is within the distribution limits [32,34,63]. The initial distribution is based on 5,000–10,000 CHO cells and was derived from experimental data using MATLAB (Generalised Extreme Value) as shown in Eq (14).


$$n_{a}(v,0)=\frac{1}{\sigma }{\left[1+ɛ(\frac{v-\mu }{\sigma })\right]}^{\frac{-1}{ɛ}-1}e^{-{\left[1+ɛ(\frac{v-\mu }{\sigma })\right]}^{\frac{-1}{ɛ}}}$$ (14)

This type of dataset on cell diameter distribution can be obtained using common automated cell counters such as the NucleoCounter ® NC-250^TM^ used herein. All the outliers in cell size, such as cell aggregates and fragments observed particularly in late days of culture were removed from the dataset. This is because it is known that the cells’ tendency to aggregate around a dead cell is higher at later stages of cell culture [64]. A careful treatment of cell volume data was required to eliminate the outliers as they might skew the cell size distribution beyond the generally reported CHO cell size [32,65]. The range was limited for diameters between 5 and 22 μm for viable cells based on our own data and previous observations [34,65]. However, the interval between 5 and 10 μm has almost no viable cells but should be added to prevent the fitted probability density function from starting at a value of almost zero volume.

#### 2.1.2 Division function

The division function reflects the increased probability of division as cell volume increases, as shown is shown in Eq (15). The division probability positive correlation with volume or age within the cell cycle is explained in the literature [66,67]. It is a function of the substrate through the addition of the growth rate. It simply means that there is no division if there is no growth. However, there are many other formulae, including discrete discontinuous, that are used in the literature [35,68–71]. The *v*_*c*_ in Eq (15) is the mean of cell volume at division, which is positively correlated to osmolality based on our data. The cell division rate for a specific mean and across a range of standard deviation values is shown in S1 Fig.


$$\Gamma (v,s)=\frac{2e^{-{\left(\frac{v-v_{c}}{ɛ}\right)}^{2}}g(v,s)}{ɛ\sqrt{\pi }[erfc(\frac{{v-v}_{c}}{ɛ})]}$$ (15)

Γ(*v*,*s*) is the cell division rate (*h*^−1^). It is important at this stage to briefly revisit the history of the division function. The division function theoretically originated from Eakman et al. who stated that “*there appears to be little conclusive evidence to support the assumption that the distribution of division mass deviates markedly from Gaussian*. *Therefore*, *it is assumed that the distribution of division mass around a division cell size mean is of a Gaussian type”* [31]. This function relies on certain assumptions such as constant extracellular environment, homogeneous mixing and exponential cell growth [55]. While it is convincing to accept that the distribution at division as a Gaussian type based on experimental measurements, several studies attempted to determine at what size a cell divides and what is the full spectrum of extracellular and intracellular drivers affecting cell volume growth [72–74].

There are many factors that affect microbial and mammalian cell volume such as cell cycle position [75,76], cell culture conditions, [77], cell cycle arrest [77], glutamine depletion [78], and many others [79,80]. To the best of our knowledge, size-based PBM has never been utilised for fed-batch CHO cell culture. Most of the aforementioned PBMs for microbial batch cultures are qualitative and ignore the effect of culture osmolality. An update is therefore suggested herein to integrate the experimentally observed shift in cell volume distribution by updating the division function. Capturing the cell volume increase would lead to better estimation of HCP volumetric secretion from dead cells by more accurately reflecting the protein content per cell.

#### 2.1.3 Partition function

The partition probability density function in Eq (16) is used to represent the asymmetric division phenomenon. [36,71]. The addition of this equation is important to capture self-similar distribution, which is the case for all cell culture system starting without imposed cell cycle arrest. This equation leads to the production of two daughter cells (i.e., *v* and *v*′−*v* at time *t*+*dt*) with a total size equal to that of their mother cell. More details with regards to the derivation of this equation can be found in the literature [81].


$$P(v,v^{\prime })=\frac{1}{\beta (q,q)}\frac{1}{v^{\prime }}{\left(\frac{v}{v^{\prime }}\right)}^{q-1}{\left(1-\frac{v}{v^{\prime }}\right)}^{q-1}$$ (16)

#### 2.1.4 Substrate and metabolite profile

The glucose and lactate profile have been coupled to capture the shift in lactate from production to consumption. Similarly, ammonia is produced from amino acid metabolism and degradation. For CHO cells and the GS-CHO cell line used herein, we can mainly attribute ammonia synthesis to asparagine and glutamine metabolism [82,83].

The following equations represent the mass balances of key amino acids, glucose, metabolites, impurities, and product. These simplified coupled equations and relationships can be found in the literature [32,82,84–86]. Asparagine is consumed at a rate given in Eq (17) with the yield *Y*_*asn*_ (*mmol cell*^−1^). All *Q* terms (*mmol* cell^−1^ *h*^−1^) in the equations below are specific consumption rates for the amino acids and glucose and production rates for the metabolites.


$$Q_{asn}={\mu_{max}f}_{lim}f_{inh}Y_{asn}$$ (17)

Ammonia is initially produced at a rate depending on asparagine consumption and consumed by the cells at later stage of the culture as shown in Eq (18). *Y*_*amm/asn*_ is the yield of ammonia from asparagine metabolism (*mmol mmol*^−1^). However, *Y*_*amm*_(*mmol cell*^−1^ *h*^−1^) is ammonia consumption rate which is triggered when its concentration increases beyond *K*_*amm*_ (3.5 *mM*) for our system.


$$Q_{amm}=Q_{asn}Y_{amm/asn}-Y_{amm}\frac{C_{amm}}{{k_{amm}+C}_{amm}}$$ (18)

In Eq (19), glucose is consumed by cell growth and maintenance-nongrowth-dependent terms [87–89]. The maintenance term, *m*_*glc*_ (*mmol cell*^−1^ *h*^−1^), is the consumption rate for glucose that does not depends on cell growth rate. Eq (20) shows lactate production from glucose in the early stages of the culture with a shift to consumption after peak viable cell density has been reached [90]. *Y*_*lac*/*glc*_ is the conversion factor for lactate from glucose (*mmol mmol*^−1^). The maintenance term of lactate *m*_*lac*_ is negative and lactate consumption does not depend on the growth rate.


$$Q_{glc}={\mu_{max}f}_{lim}f_{inh}Y_{glc}+m_{\begin{array}{c} glc \end{array}}$$ (19)

$$Q_{lac}=Q_{glc}Y_{lac/glc}-m_{lac}$$ (20)

The rest of the amino acids are produced such as alanine, glutamine and consumed such as glutamate which follow a simpler kinetics that depends on growth rate as shown in Eqs (21)–(23), respectively. The yields in these equation *Y*_*ala*_, *Y*_*gln*_ and *Y*_*glu*_ (*mmol cell*^−1^) and *Q* (*mmol cell*^−1^ *h*^−1^).


$$Q_{ala}={\mu_{max}f}_{lim}f_{inh}Y_{ala}$$ (21)

$$Q_{gln}={\mu_{max}f}_{lim}f_{inh}Y_{gln}$$ (22)

$$Q_{glu}={\mu_{max}f}_{lim}f_{inh}Y_{glu}$$ (23)

The following mass balances equations are used to calculate the concentration of the substrates and metabolites including the salts which were used to calculate osmolality. They show a single parameter to account for a consumption or a production rate.


$$\frac{d[V\;C_{asn}]}{dt}=-Q_{asn}V\;N+F_{in}\;C_{Feed-asn}-C_{asn}F_{out}$$ (24)

$$\frac{d[{V\;C}_{amm}]}{dt}=Q_{amm}V\;N-C_{amm}F_{out}$$ (25)

$$\frac{d[V\;C_{lac}]}{dt}=Q_{lac}V\;N-C_{lac}F_{out}$$ (26)

$$\frac{d[{V\;C}_{glc}]}{dt}={-Q}_{glc}V\;N+F_{in}C_{Feed-glc}-C_{glc}F_{out}$$ (27)

$$\frac{d[V\;C_{gln}]}{dt}=Q_{gln}V\;N-C_{gln}F_{out}$$ (28)

$$\frac{d[{V\;C}_{glu}]}{dt}={-Q}_{glu}V\;N+F_{in}C_{Feed-glu}-C_{glu}F_{out}$$ (29)

$$\frac{d[V\;C_{ala}]}{dt}={-Q}_{ala}V\;N-C_{ala}F_{out}$$ (30)

$$\frac{d[V\;C_{Na}]}{dt}=-Q_{Na}V\;N-C_{Na}F_{out}$$ (31)

$$\frac{d[V\;C_{K}]}{dt}=-C_{K}F_{out}$$ (32)

#### 2.1.5 mAb and host cell proteins

Our experimental data support that specific mAb productivity is constant and independent of cell growth rate, as shown in Eq (34). This reduces the number of used parameters and allows for direct comparison of *m*_*mAb*_ under the two culture temperatures.


$$Q_{mAbs}=m_{mAb}$$ (33)

$$\frac{d[V\;mAb]}{dt}=Q_{mAb}V\;N-{C_{mAb}F}_{out}$$ (34)

Based on our experimental data, we hypothesise that there are two sources of HCPs, one being secretion from viable cells (at a constant rate of *q*_*HCP*_ in *g cell*^−1^ *h*^−1^) and the second being lysis of dead cells (at a constant volumetric rate of *q*_*HCP*(2)_) as shown in Eq (35). The cell volume is taken into account in the prediction of HCP release from dead cells by the inclusion of the average normalised cell volume $\bar{v}$ in Eq (35). As cells increase in volume, the host cell protein content per cell also increases. To account for this, we consider *q*_*HCP*(2)_ on a per unit cell volume basis, such that, when multiplied by the cell volume, we obtain a more accurate estimate of released HCPs. Measuring the proportion of secreted and non-secreted HCPs experimentally is very challenging. As these proteins degrade during cell culture through the proteolytic activity of certain HCPs, ELISA measurements could also lead to underestimation of HCP concentration [91]. The release of intracellular content from a dead cell (i.e., DNA and HCPs) does not seem to be an immediate process and depends on the physical properties of the bioreactor, cell size and cell membrane fluidity and resistance to shear stress. Pluronic F68 is known to reduce the plasma-membrane fluidity of cells and this has been suggested as a possible mechanism of protection against shear force [92,93]. For the purpose of this model, we have chosen to neglect HCP degradation in the supernatant due to the inability to accurately determine the associated rate from existing experimental data.


$$\frac{d[V\;HCP]}{dt}=q_{HCP}V\;N+q_{HCP(2)}V\;\bar{v}(N_{l}+N_{d})$$ (35)

#### 2.1.6 Temperature shift

There are three different ways to describe the effect of variation in pH and temperature on cell culture dynamics. Firstly, obtaining separate sets of parameter values each for a different set of culture conditions, which is adopted herein. The second method is to use a continuous function for a wider design space [94,95], e.g. by describing maximum cell growth rate as a continuous function of temperature using the Arrhenius and Eyring equations [96]. The third method is based on statistical modelling to derive a correlation from experimental data or other common equation for the pH such as Henderson-Hasselbalch [89,97,98].

#### 2.1.7 Culture osmolality

The data from the four bioreactor runs were used to formulate an equation that can estimate the osmolality of CHO cell culture based on the values of certain key extracellular components that are routinely measured, namely glucose, lactate, Na^+^ and K^+^. Osmolality (*Osm*, in mOsm kg^-1^) is approximated by Eq (36) which has three parameters determined using the experimental datasets. Eq (36) is only applicable for our commercially available CHO cell culture medium; however, the same approach can be used to form an equation of osmolality for other systems. The terms *α*_1_, *α*_2_ and *α*_3_ equal 1.48, 0.70 and 2.18 respectively with unit of mOsm kg^-1^ mM^-1^. Many other similar osmolality equations can be found in the literature [99].


$$Osm=\alpha_{1}{(C}_{Glc})+\alpha_{2}(C_{Lac})+\alpha_{3}(C_{{Na}^{+}}+C_{K^{+}})$$ (36)

The mean absolute percentage error (MAPE) is 2.5%, which is deemed acceptable [100].

### 2.2 Experimental materials and methods

#### 2.2.1 Bioreactor operation

Briefly, CHO GS46 producing chimeric IgG_4_ antibody (Lonza Biologics) was revived and cultured in shake flasks (Corning, NY) of CD CHO medium (Life Technologies, UK) and shaken at 140 RPM as described in [11,101]. The selective agent MTX was added to the revived cells and the first subculture. Cells were subcultured in fresh medium every 4 days at a seeding density of 2 × 10^5^ cells/mL. Then, bioreactor (Applikon Biotechnology, Schiedam, the Netherlands) was inoculated at a seeding density of 3 × 10^5^ cells/mL with an initial cell culture volume of 1.2 L. The temperature, pH and dissolved oxygen were controlled at 36.5°C, 7.0 and 50% respectively. The bioreactor was supplemented with CD EfficientFeed™ C AGT™ (Life Technologies, UK) at 10% cell culture volume on alternate days starting from 2^nd^ day. Two of the bioreactors were kept at 36.5°C (sometimes refer to as 37°C or physiological temperature in this work) whereas the other two were shifted to 32°C on the 5^th^ day. The cell density and diameter were measured daily using NucleoView NC-250 (ChemoMetec, Denmark). All cells below 5 and greater than 22 μm were removed as they do not represent the viable CHO cells but outliers as a result of cell aggregation and fragmentation [65,102]. The cell volume was calculated based on the diameter value assuming a spherical cell shape. The cell volume was normalised to represent values from 0 to 1 cell volume domain. The Distribution Fitter tool of MATLAB (R2017B) was used to generate the probability density function of that cell volume distribution.

#### 2.2.2 Extracellular metabolite analysis

The protocol is taken from Sellick et al. metabolite profiling study [103,104]. The procedure is as follow:

*Sample derivatisation*. 20 μL of the cell culture supernatant was withdrawn from a fully thawed 1 mL sample tube and mixed with 200 μL of (water/methanol/isopropanol at a volume ratio of 2/5/2). The tube containing the supernatant was placed in a vacuum rotary centrifuge at 30°C for 30 minutes to dry out completely until a metabolite pellet was observed at the bottom of the tube. The dried pellet was derivatised by addition of 10 μL methoxamine hydrochloride (MOX, 40 mg/mL in pyridine). The sample was incubated for 90 minutes at 30°C with gentle shaking. The sample was then trimethylsilated by addition of 90 μL N-methyl-N-(trimethylsilyl) trifluoroacetamide (MSTFA) containing 1% (v/v) trichloromethylsilane (TMCS). The sample was then incubated at 37°C for 30 minutes. The derivatised sample was cooled to room temperature and 70 μL was pipetted in silanised GC vials (National Scientific).

*GS-MS analysis*. The analysis was performed in a 7890A GC system (Agilent Technologies) coupled to a 5975C Inter XL MSD with Triple-Axis Detector (Agilent Technologies). The injected sample was 1 μl with 10:1 split ratio on a DB-5MS + DG column (Agilent Technologies; 250 μm × 30 m × 0.25 μm thickness with 10 m DuraGuard) with helium as a carrier gas. Analytes were separated by isothermal chromatography at 60°C for a minute, then increased at a rate of 10°C per minutes to approach 325°C and remain at this value for further 10 minutes. The temperatures of the injector, MS source and MS quad were set at 250, 230 and 150°C respectively. The analyte peaks in the raw chromatograms were identified using MSD ChemStation (Agilent Technologies) to search the Agilent Fiehn GC/MS. Identification was based on the RTs and fragmentation patterns. Peak areas were determined with reference to myristic acid as an internal standard for all amino acids.

*Analysis of other metabolites*. BioProfile FLEX (Nova Biomedical, MA, USA) was used to measure the concentrations of glucose, lactate, glutamine, glutamate and ammonia in cell culture supernatants. The instrument performed automated enzymatic assays on glucose, lactate, glutamine, and glutamate, while ammonia concentration was measured by electrochemical means with a phosphate assay.

#### 2.2.3 mAb titre

The concentration of the mAb product was measured using the Blitz system (Pall ForteBio Europe, Portsmouth, UK). The biosensor was first hydrated in sample diluent (Pall ForteBio Europe, Portsmouth, UK) for 30 minutes and then locked onto the BLItz instrument. 4μL of the sample were pipetted onto the sample holder and analysed. For each run, the binding rate was measured for 60s and concentration of the sample was directly proportional to the binding rate. A standard curve of mAb concentration was plotted against the binding rate to interpolate the corresponding titre.

#### 2.2.4 Osmolality

The osmolality of the supernatant was measured by Osmomat 3000 (Gonotec, Berlin, Germany) based on freezing point. The equipment was calibrated with 0, 300 and 800 mOsm kg^-1^ solutions and sample sizes were 50 μL each in 500 μL measuring vessels (Gonotec, Berlin, Germany). Duplicate measurements were taken for each sample.

#### 2.2.5 LDH and DNA analysis

Lactate dehydrogenase (LDH) is used to identify and quantify dead cell population [105]. The Pierce™ LDH Cytotoxicity Assay Kit (Thermo Scientific™) was used to quantify the cell lysis rate. Cell culture supernatant samples were placed in 96-well flat bottom plates. Firstly, a serial dilution of cells was made from 2×10^5^ to 2.5×10^4^ cells per mL in 100 μL triplicate wells including medium to measure background activity After that, 10 μL lysis buffer was added to all wells including cell culture supernatant samples and incubated at 37°C for 45 minutes.

50 μL of each well was transferred to a new plate and mixed with 50 μL Reaction Mixture. After a 30-minute room temperature dark incubation, reactions were stopped by adding 50 μL Stop Solution to each well. Absorbance at 490 nm and 680 nm were measured using a plate-reading spectrophotometer to determine each sample LDH activity. The absorbance reading at 680 was subtracted from that at 490 to eliminate any background signal from the equipment.

Quant-iT™ PicoGreen™ dsDNA Assay Kit (Invitrogen™) was used to quantify double stranded DNA in the supernatant. Calibration curve of Lambda DNA was made in the range from 0 to 1 μg/mL which is described as high-range standard curve in the protocol. Every 96-well microplate included samples to construct the calibration curve and three technical replicates. The volume of the sample was 200 μL which was mixed with same volume of Diluted Quan-iT™ PicoGreen™ Reagent and incubated for 5 minutes at room temperature in dark. Fluorescence was then measured in a microplate reader (excitation at 485 nm, emission at 520 nm).

#### 2.2.6 Cell volume data analysis

Plotting the average cell size as a function of osmolality illustrates the effect of osmolality on cell volume. Outliers in cell size, such as cell aggregates and fragments observed particularly in late-stage culture, were removed from the data. The range was limited to diameters between 10 and 20 μm based on previous observations [32,65]. The approximated non-linear function, Eq (37) was obtained using the curve fitting option in OriginLab, in which *y* is the average cell volume or SD of cell volume and *x* is the osmolality. The same equation can be used to correlate the standard deviation of the cell volume as a function of osmolality and both sets of parameter values are given in S1 Table. The best fit was given by dose response and Boltzmann function in both cases. Dose dependence of osmolality effects on many cellular variables has been already mentioned in the literature [34,65,106].


$$y=A_{1}+\frac{{(A}_{2}{-A}_{1})}{\left(1+10^{\left((x_{0}-x)\times p\right)}\right)}$$ (37)

Regardless of the bioreactor temperature, the size growth evolution follows a similar trend. However, although there appears to be a correlation between osmolality and cell volume, this is not sufficient to fully describe the data presented herein. It is important to highlight the fact that the variation in cell volume is narrower for the biological replicates but utilising the two temperatures datasets is crucial to cover the wider range of volume distribution.

### 2.3 Parameter estimation

The model equations are solved in the gPROMS 5.0. 1 model building environment (Process Systems Enterprise, www.psenterprise.com/products/gproms, 1997–2020). Our PBM was solved by backward finite difference method (BFDM), which is known to be appropriate this type of integral, partial differential, and algebraic equations (IPDAEs) systems [107]. In this study, parameters estimation is obtained by the maximum likelihood method from gPROMS which attempts to determine values for the uncertain physical and variance model parameters that maximise the probability that the model will predict the experimentally measured values. The statistical constant and linear variance models were used. The 95% confidence intervals for estimated parameters were deemed satisfactory at 10% of the final parameter values [108].

## 3 Results and discussion

### 3.1 The PBM accurately describes the experimental data

**Fig 1:**
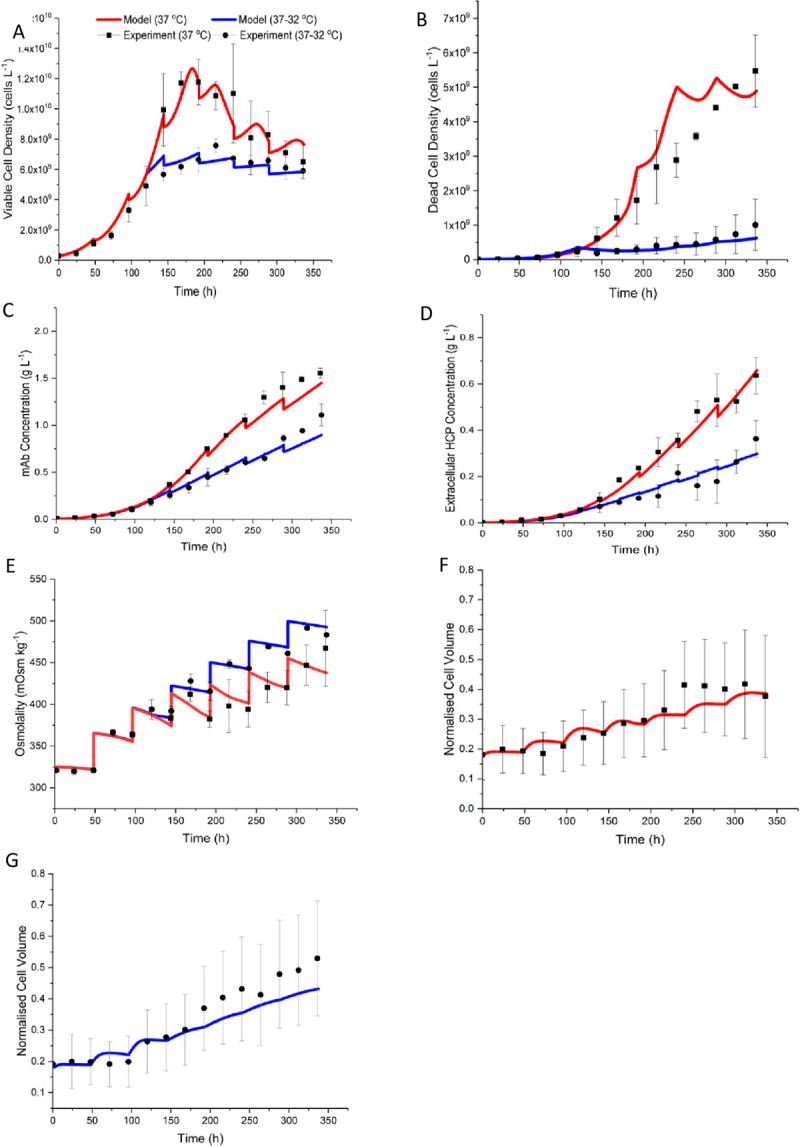
Comparison of model simulation results with experimental data. The red and blue lines represent the model output for the physiological and mild hypothermic bioreactors, respectively. The experimental data are represented in square (physiological temperature) and circular (mild hypothermia) points for (A) viable cell density; (B) dead cell density (C) mAb titre, (D) extracellular HCP concentration, (E) culture osmolality, (F) and (G) normalised cell volume for physiological temperature and mild hypothermic case, respectively. Error bars represent one standard deviation.

The model simulation results are compared with experimental data for the physiological temperature and mild hypothermic culture conditions in Fig 1. The viable cell density in Fig 1A is well captured even during the last a few days of the culture when kinetic models typically fail to capture the decline in cell growth rate in the presence of nutrients. The reduction in viable and dead cell densities as temperature was decreased is also captured well. However, the agreement for dead cells in Fig 1B is less satisfactory for the physiological case as the cell lysis rate might not be constant during the culture potentially due to the extracellular accumulation of intracellular components that may promote faster degradation. At the lower temperature, the dead cell profile has very large standard deviation in the second half of the culture, signifying the high biological variability in quantifying dead cells in comparison to viable ones. It is worth mentioning that the cell death rate is a non-linear function of the growth rate. Although several other cell death formulae can be used, the selected one requires fewer parameters to be estimated. The disadvantage is that the reduction in cell growth rate is not always matched by an increase in dead cell population.

**Fig 2:**
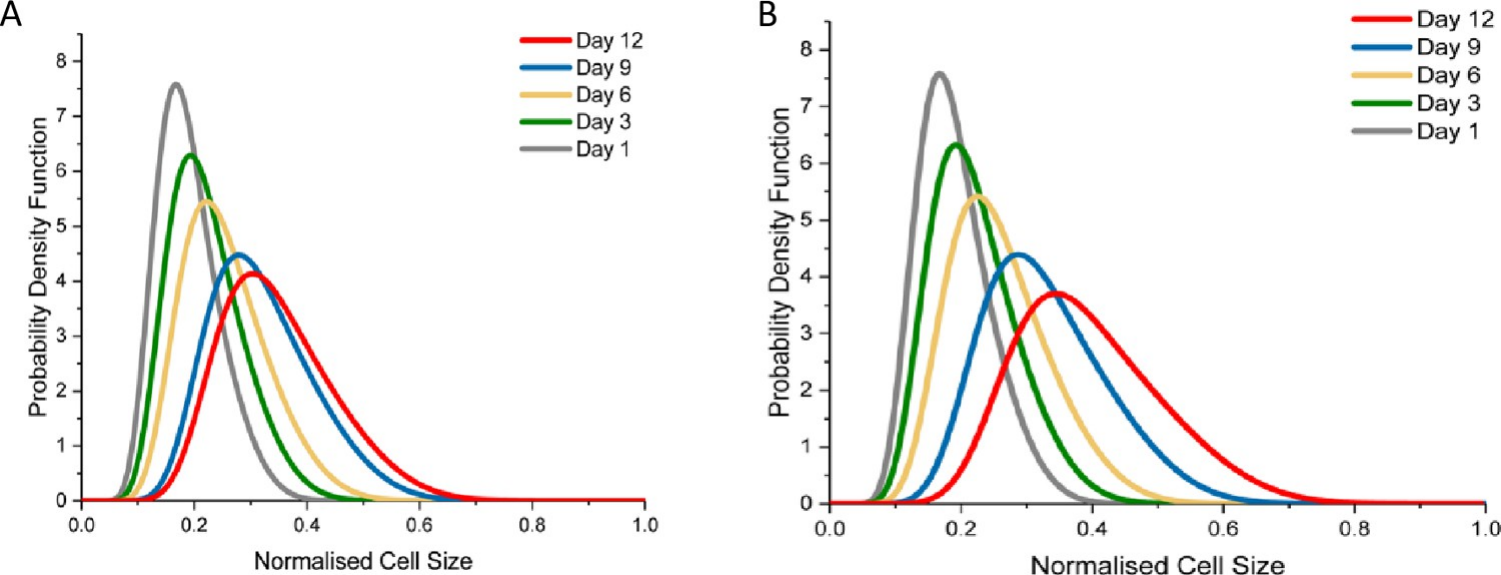
Normalised cell distribution for the physiological temperature (A) and mild hypothermic cultures (B). The cell diameters were taken from the NucleoCunter™ and the given distributions are obtained from distribution fitting in MATLAB.

The mAb and HCP concentrations are captured well as shown in Fig 1C and 1D. In the case of mAb concentration, there is some discrepancy in the final days of culture, which may be due to product release during cell death. The model accurately describes HCP accumulation, although there is increased variability in HCP measurement in late-stage culture, which is in line with the biological variability evident in dead cell density measurements. Culture osmolality is higher for the mild hypothermic case in Fig 1E because the feeding regime is the same even though the cells are less metabolically active. This leads to an accumulation of overfed nutrients extracellularly. The simulation results for the remaining metabolites considered by the model, i.e., asparagine, ammonia, glutamate, glutamine and alanine, are compared to experimental data in S2 Fig. The normalised cell volume increase is shown in Fig 1F and 1G for the physiological and mild hypothermic temperatures, respectively. The significant increase in the experimentally determined values for cell volume around 250 h is believed to be due to cell aggregation. Aggregation is known to increase in the last a few days when sticky intracellular components are released from dead cells [109]. Overall, the agreement between the model simulation results and experimental data is satisfactory but can be improved if the parameter estimation strategy focused on fitting the distribution rather than the average size. The volume distributions are shown in Fig 2A and 2B for the physiological and hypothermic cases, respectively. As the culture conditions for two sets of bioreactor runs are identical until mild hypothermia is introduced, the volume distributions are highly comparable initially. In both cases there is a gradual increase in cell size, but this is more pronounced in in the cultures grown at 32°C due to the larger increase in culture osmolality.

According to Nolan and Lee, there are six characteristics defining an ideal cell culture model [105]. One of those is being able to describe long term dynamics of the fed-batch culture. It is very challenging because a deviation between simulation results and experimental data is commonly observed in the last days of the fed-batch culture. This has led researchers to explore cell culture phase segregation to look at cell growth dynamics at these different culture phases separately [32]. The discrepancy could be because there are many variations in extracellular components such as vitamins, nucleotides, growth factors, folic acid, which are not routinely measured and less simple to account for in a model [83]. Moreover, many phenomena occur in the last days of cell culture such as cell fragmentation and lysis, and degradation of proteins. Additionally, gene regulation events may lead to less traceable phenotypes. This has also been observed by Munzer et al. who found a greater deviation in cell cycle phase prediction in late-stage culture [110].

### 3.2 Understanding the origin of extracellular HCPs

There are many factors that affect HCP accumulation and degradation such as culture temperature and pH [111]. Using the model presented above, we therefore sought to understand what proportion of HCPs is secreted versus released upon dead cell lysis. Given that most staining dyes do not quantify the cells that have already lysed after death, the commonly used method of trypan blue dye exclusion for determining culture viability leads to underestimation of cell death. For this reason, we employed orthogonal methods for determining cell content in the supernatant. Specifically, we measured lactate dehydrogenase (LDH) and DNA, which are released from cells with compromised membrane [112]. LDH has been used to determine necrotic cells as leakage occurs of plasma membrane [113].

**Fig 3:**
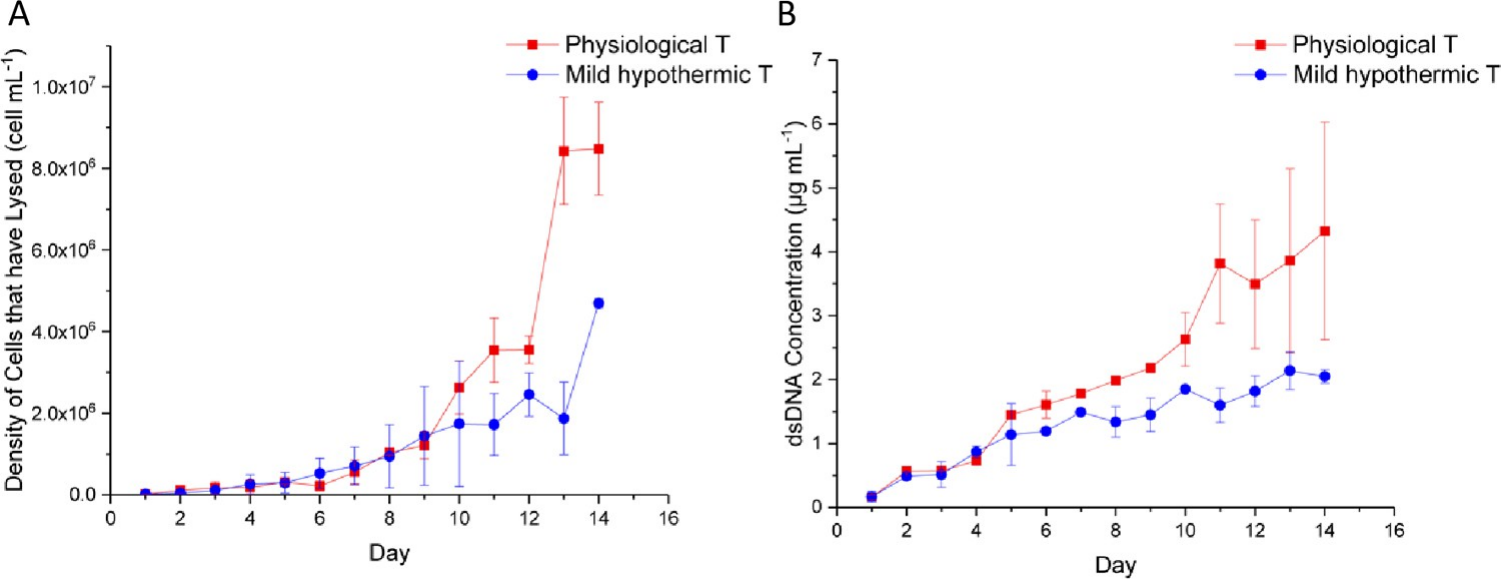
(A) Concentration of cells that have lysed as inferred from the results of the LDH assay; (B) Double stranded DNA concentration in the supernatant.

From a first glance at Fig 3, extracellular LDH and DNA are higher at the physiological temperature in comparison to mild hypothermic case. This is expected as dead cell density is significantly higher at physiological temperature as measured by NC-250 in Fig 1B. For both markers, the deviation between the two bioreactors increases at a higher rate after day 10.

The dead cell population (Fig 1B) increases linearly after day 5 under physiological temperature but at a much slower rate under mild hypothermia. Although this trend is observed in the DNA profile (Fig 3B) and HCP concentration (Fig 1D), this is not the case for LDH concentration until day 10. Similarly, the large increase in LDH on days 13 and 14 is not observed in the DNA or dead cell concentration profiles. More importantly, the dead cell population determined by NC-250 is five times higher for the physiological temperature bioreactor, which is not reflected in any of the values of DNA, HCP concentration apart from day 13 for LDH. The number of lysed cells estimated based on LDH quantification (Fig 3A) is approximately 60% higher than the dead cell density measured by NC-250 for the physiological temperature bioreactor runs. Interestingly, the LDH assay identified about 400% more lysed cells than dead cells on day 14 for the mild hypothermia case. Comparing the concentrations of DNA, HCP and LDH between physiological and hypothermic bioreactor runs, we see that the former are approximately two times higher than the latter for all markers. This may be due to the degradation rate of these molecules being higher at 37°C. The degradation rate of LDH in the supernatant is known to be higher at 37°C in comparison to 32°C [114] and has been reported to be double that of DNA [111,115]. The degradation of HCP is also expected to be reduced at lower temperatures [116].

It is also important to look at the HCP profile which, although following the same trend with the loss of culture viability, does not correlate as well with dead cell density. It is worth noting that viable cell density at physiological temperature is double that at mild hypothermia. This could also support the hypothesis that when culture viability is high, most HCPs are secreted as noted in other studies [117–119].

It has been reported that the DNA content of a CHO cell is 1.11 pg [111], while reports of the intracellular protein content of CHO cells vary in the literature. For example, Pan et al. reported an average of 71 pg cell^-1^, while Cheung reported a value of 82 pg cell^-1^ [120], Kol et al. of 150 pg cell^-1^ proteins [121] and Chaudhuri of a range from 5 to 40 pg cell^-1^ [122] depending on the culture day. The Kol et al. value was eliminated as it is based on cells that were harvested on day 7 when culture viability was less than 90%, which is not representative of our system. The other two values were averaged and the resulting value, 75 pg cell^-1^, was used in subsequent analysis.

As the DNA degradation rate is smaller than that of LDH and it is more stable than proteins, it was used to quantify cells that released their intracellular contents in this study. These are around 3.6 and 1.8 million cells mL^-1^ for physiological and mild hypothermic conditions on the final day, respectively, based on Fig 3B. If all intracellular content was fully released and assuming that the protein content per cell and extracellular degradation rate at the two temperatures are the same, then, 0.27 and 0.13 mg mL^-1^ come from the dead cells, at physiological and mild hypothermic bioreactors, respectively. The difference between these estimates and the measured concentrations (Fig 1D) is expected to have been secreted. This means that slightly more than half of the total HCPs at the end of the culture are, in fact, secreted. This is supported by the a recent study by Kol et al., who achieved a reduction of more than 50% in HCP concentration for high viability cell cultures >90% by knocking out 6 HCPs genes [121].

We used the estimates of the model parameterization exercise to further inform our analysis (parameter estimates and confidence intervals are shown in S2 Table). Because of the significant discrepancies between the measured dead cell density and cell lysis estimates derived from LDH and double stranded DNA measurements, we estimated the cell lysis rate, *k*_*l*_, in Eq (7) based on the viable and dead cell concentration data only.

The initial guess and upper and lower bounds for the two HCP parameters were informed by the specific HCP secretion rate from viable cells estimated above and the total protein content per cell that is released to the extracellular matrix upon cell death (as discussed above). Different secretion rates from viable cells are estimated, based on the parameter estimation (S2 Table), for the two culture temperatures: 2.4×10^−11^ *g cell*^−1^ *h*^−1^ at physiological temperature and 3.4×10^−14^ *g cell*^−1^ *h*^−1^ for mild hypothermic temperature. This could mean that metabolically active cells at higher cell density secrete HCPs at a higher rate for biologically known purposes such as for cell signaling. Considering the higher dead cell density at 36.5°C, a higher proportion of extracellular HCP concentration is estimated to be released from non-viable cells. However, based on the estimated value of the HCP production rate from dead and lysed cells, mild hypothermia seems to either increase cellular HCP content or slow down the degradation of HCP in comparison to physiological temperature, S2 Table. It should be noted that the release and degradation of HCPs after death is expected to proceed at a higher rate at physiological temperature, assuming that the dead cell density at both temperatures has been accurately determined. It is worth mentioning that many approaches for estimating *q*_*HCP*_ and *q*_*HCP*(2)_ were explored such as simultaneous estimation of the parameters and one parameter at a time. Our estimated values give the best fitting quality considering the HCP profile. It appears difficult to draw any firm conclusions without experimentally establishing the degradation rate of HCPs at the different temperatures and how culture temperature affects the intracellular HCP content of CHO cells. Another challenge stems from the fact that HCPs have a wide range of half-lives [123,124]. Nonetheless, our analysis shows that the assumption that extracellular HCPs mostly derive from dead cells, as various publications claim [125], can only hold true for culture viability values well below 80%.

#### 3.3 Using the model to perform *in silico* optimisation

The trained model has been used to maximise different objective functions with certain technical and economical constraints applied to determine possible process operation scenarios. In this optimisation problem four scenarios were looked at as summarised in Table 1. Physiological temperature growth scenarios are given in the first two rows and the temperature shift scenarios follow. Cases 1 and 4 are the control experiments (standard cases) for the physiological and mild hypothermic conditions, respectively, which were used for parameter estimation only. No feed is introduced in the first 48 hours of culture in line with the feeding regime used for model parameterisation. The minimum acceptable viability in this case is 80%, which reflects common industrial practice as cell death leads to release of cell-derived impurities such as DNA, HCP and product with immature glycosylation [126].

**Table 1:** A summary of mAb optimisation scenarios.

Case	Description
**2**	**Max [mAb],** subject to: * Viability ≥ 80%, * Feed in = 0, t ≤ 48 h, * Control variable ∈ [10,40], * 1.1 L ≤ V ≤1.3 L, * 0 ≤ Feed in ≤120 mL/day, * Feed out = 55 mL/day.
**3**	Max [mAb/HCP] * The same previous constraints are applied.
**5**	**Max [mAb]**, with the same constraints of Case 2 * The temperature switch time point (found to be 140 hours)
**6**	Max [mAb/HCP] * The same constraints are applied in addition to the temperature switch time point (found to be 100 hours)

Feeding of all amino acids is recommended to maintain consistent nutrient availability. This strategy is implemented to achieve higher cell density and protein production [127–129]. The concentrations of amino acids and glucose are not set as degrees of freedom, unlike in Kappatou et al. [126], as the model has been verified with data from processes using comercially available feed and extrapolation to different conditions may not be valid. Separating glucose and growth-limiting amino acids form the rest of the nutrients might be recommended as shown in the study by Xing et al. [4]. However, this was not considered in this study.

**Fig 4:**
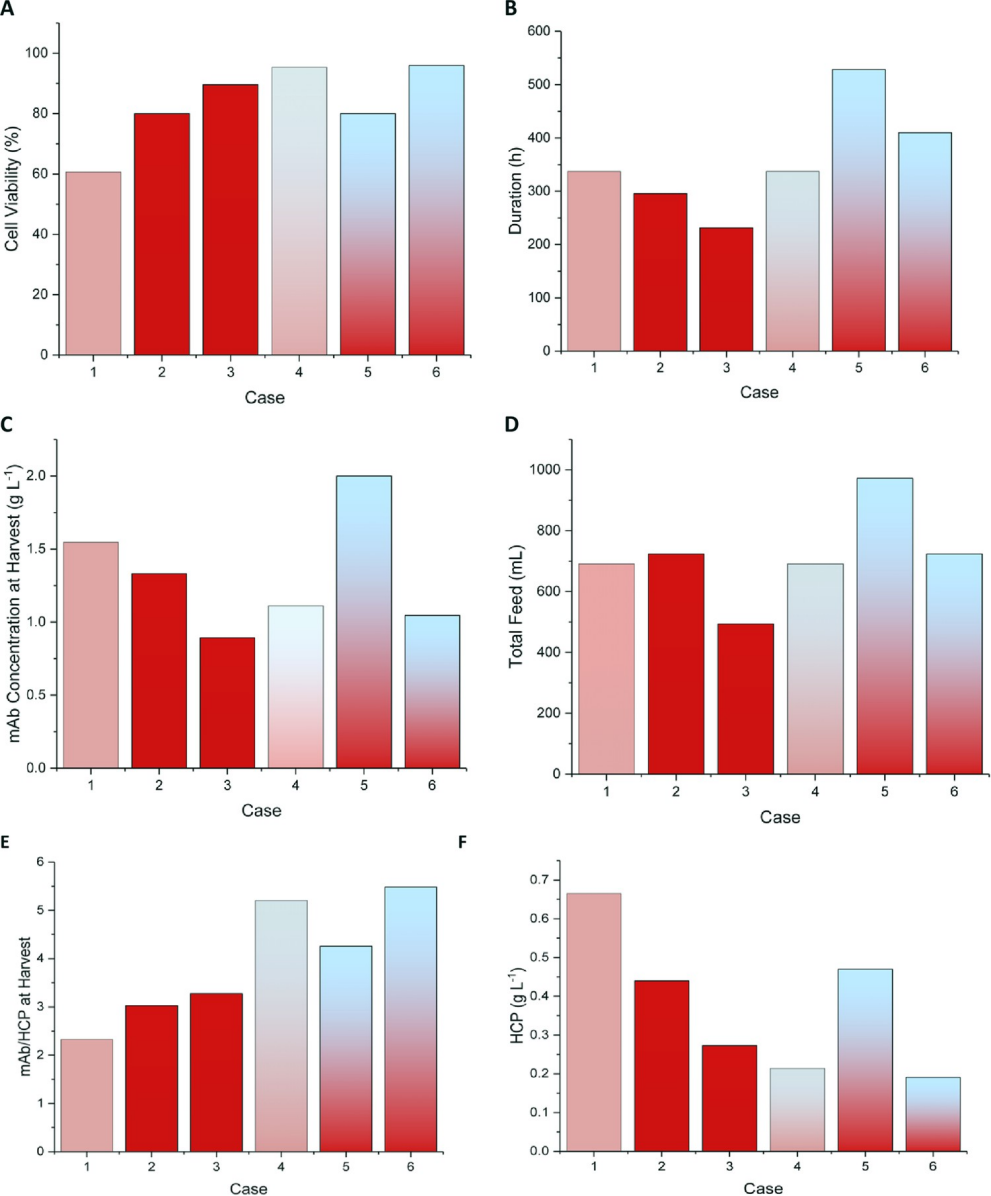
Results for the six optimisation scenarios shown in Table 1 for: (A) culture viability, (B) bioreactor run duration, (C) mAb concentration at harvest, (D) total feed input, (E) mAb/HCP concentration ratio, (F) HCP concentration at harvest. Cases 1 and 4 are the control experiments (standard cases) at physiological and mild hypothermic conditions, respectively, which were used for parameter estimation. The first three cases are for cultures grown under physiological temperature whereas cases 4–6 are cultures operated with a shift to mild hypothermia. The optimum temperature downshift time is after 140 h and 100 h for cases 5 and 6, respectively.

The outcome of the optimisation studies is compared to the respective control experiment in Fig 4. The 80% culture viability constraint has not been violated for any of the optimised cases (Fig 4A) but cultures at mild hypothermic conditions (cases 5 and 6) sustain a higher viability for longer compared to those at physiological temperature (cases 2 and 3), as shown in Fig 4B. The 80% viability constraint results in earlier harvest time for cases 2 and 3 (Fig 4C).

For the mild hypothermia case, the titre increase is related to the relatively longer culture duration when the objective of the optimization strategy is to maximise mAb titre. However, if product/impurity ratio is chosen as the objective function, the culture duration is slightly reduced to achieve higher viability and product:HCP concentration ratio. If the chromatography columns are designed to withstand relatively high impurities feedstock, then case 5 seems the best choice. Otherwise, case 6 might be more attractive, but more financial assessments should be done in the downstream unit operations to evaluate the lifetime and cost associated to Protein A chromatography column was and regeneration frequency. Finally, Fig 4E and 4F depict the effect of changing the objective function to include the impurity concentration as a decision criterion.

The time of the temperature downshift is critical as cell growth is significantly slower at reduced temperatures. This negatively affects the attainable viable cell density and hence the mAb titre. However, mild hypothermia offers a culture duration extension to compensate the drop in titre, reduced consumption of nutrients and decreased accumulation of waste products [130,131]. Nolan and Lee looked at several scenarios such as feeding strategy, day of temperature shift, initial seeding density. Based on their study, the effect of lower seeding density is similar to early temperature shift in affecting viable cell density and suppress the lactate shift [105].

## 4 Conclusion

This study shows the usefulness of mathematical modelling for exploring trade-offs in bioprocess performance. Integrating this model with a downstream purification model to evaluate the cost of removing these fractions of impurities, can help determine what concentration of HCPs can be economically tolerated in the cell culture supernatant and aid whole bioprocess design. While the PBM was improved by including the cell volume increase which can be readily implemented in future studies, the content of HCPs per unit cell volume at the two growth temperatures should be identified experimentally to improve the quantification of secreted and non-secreted HCP production and their degradation rate in the supernatant at different cell culture temperatures.

## Supporting information

S1 Fig Division function for a mean of 0.5 and three different SD. (DOCX)

S2 Fig The model fitting for the main metabolites and substrate for the physiological (dark red) and mild hypothermia (light red). The black and grey experimental data correspond to the physiological and mild hypothermia respectively. Glucose(A), Lactate(B), Asparagine(C), Ammonia(D), Glutamate(E) Glutamine(F) Alanine(G).
(DOCX)

S1 Table Parameter values for Eq (37). (DOCX)

S2 Table Parameter values with their 95% confidence interval and units. (DOCX)
