# Supplementary figures and images for: Population balance modelling captures host cell protein dynamics in CHO cell cultures
Source: PLoS One. 2022 Mar 23;17(3):e0265886. doi: 10.1371/journal.pone.0265886 (PMC8959726; doi:10.1371/journal.pone.0265886)

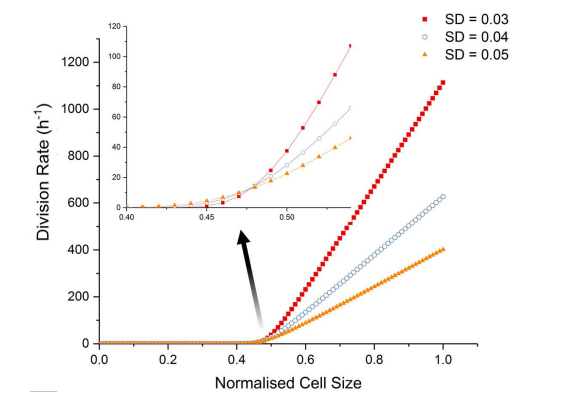


**Figure S1**: Division function for a mean of 0.5 and three different SD.

Supplement: S1 Fig — (DOCX) [file pone.0265886.s001.docx]
