# Supplementary material for: Population balance modelling captures host cell protein dynamics in CHO cell cultures
Source: PLoS One. 2022 Mar 23;17(3):e0265886. doi: 10.1371/journal.pone.0265886 (PMC8959726; doi:10.1371/journal.pone.0265886)
Supplement: S2 Fig — The model fitting for the main metabolites and substrate for the physiological (dark red) and mild hypothermia (light red). The black and grey experimental data correspond to the physiological and mild hypothermia respectively. Glucose(A), Lactate(B), Asparagine(C), Ammonia(D), Glutamate(E) Glutamine(F) Alanine(G). (DOCX) [file pone.0265886.s002.docx]

| **A**  **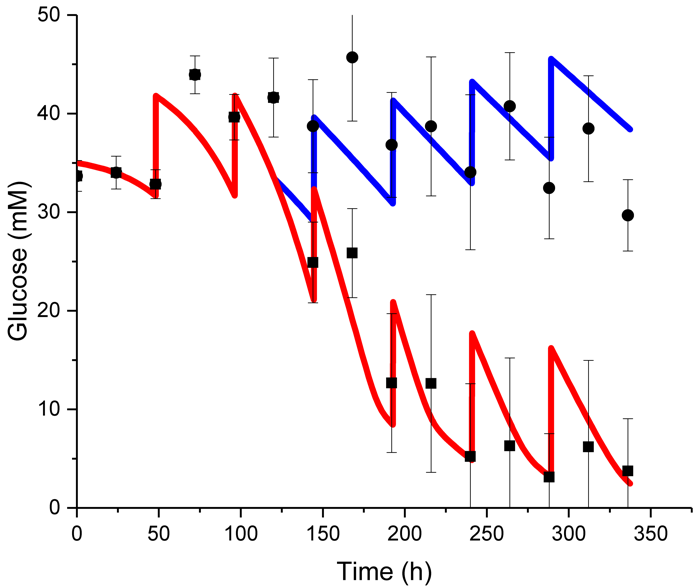** | **B**  **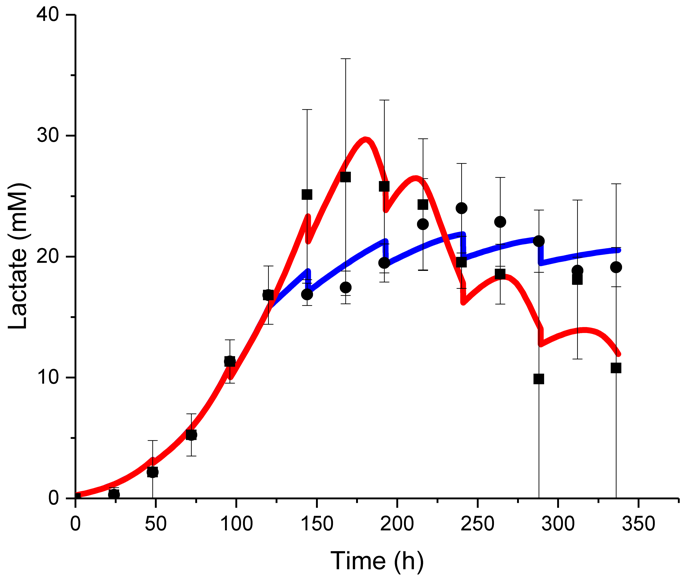** |
| --- | --- |
| **C**  **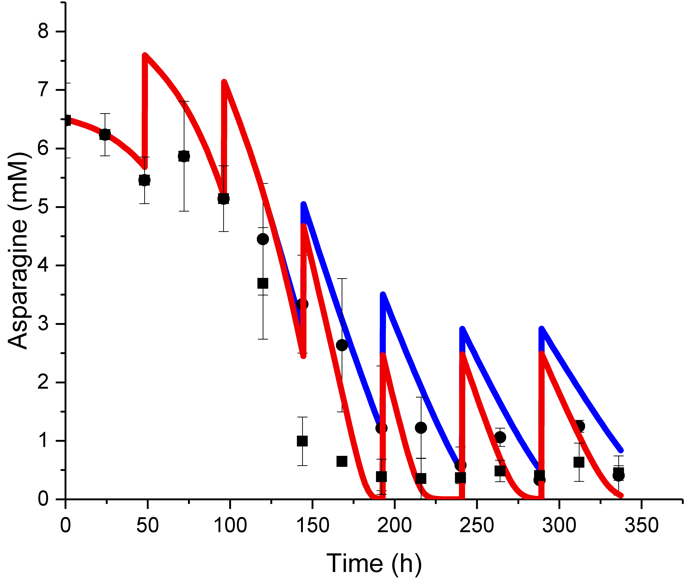** | **D**  **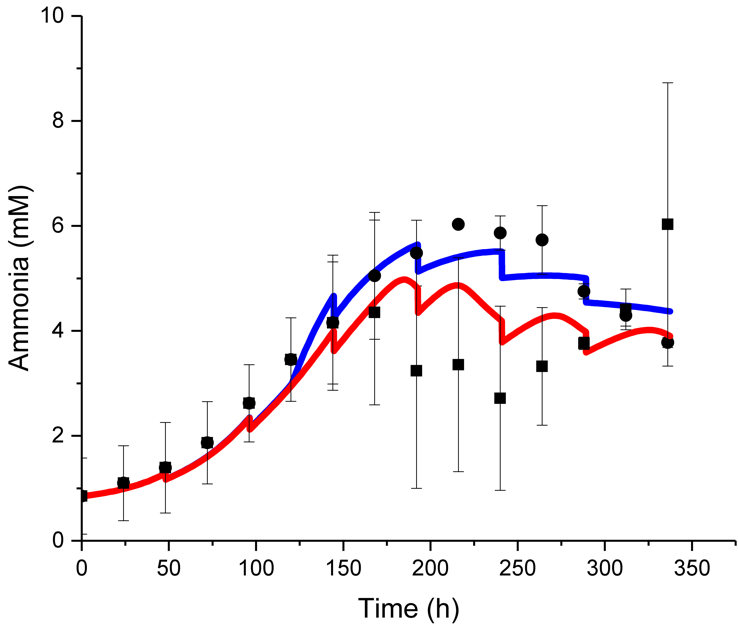** |
| **E**  **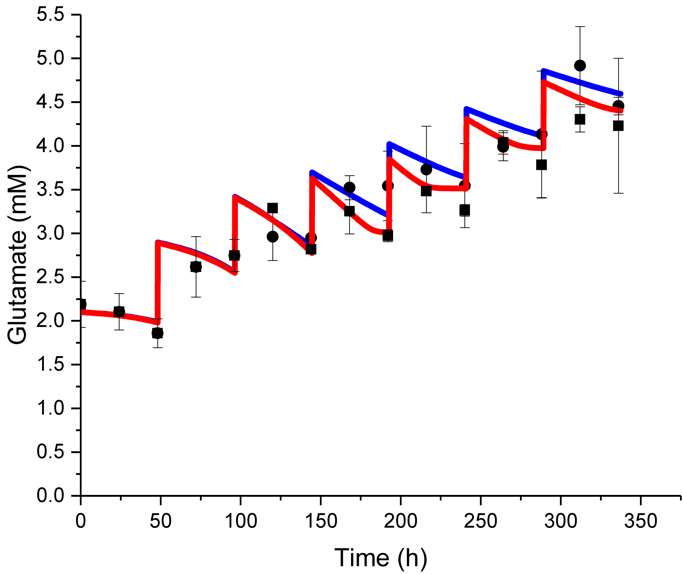** | **F**  **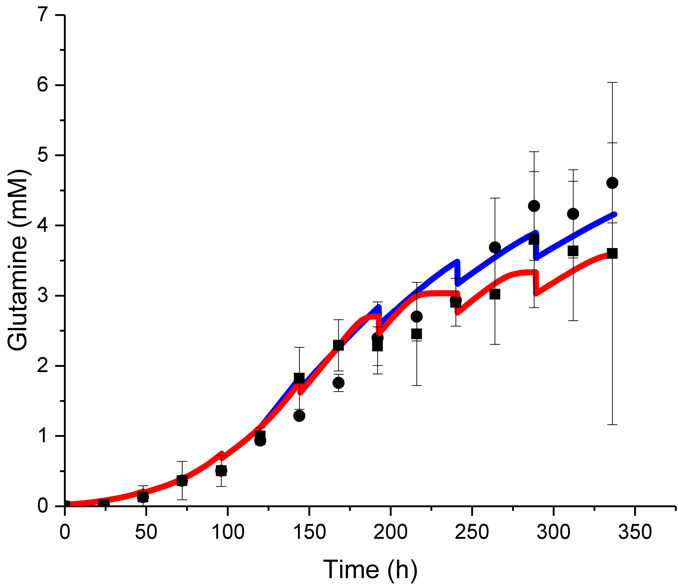** |
| **G**  **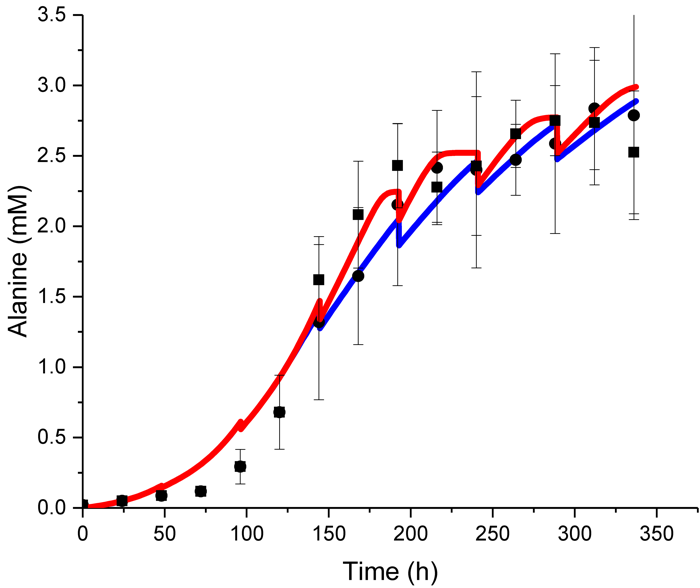** | **Figure S2**: The model fitting for the main metabolites and substrate for the physiological (dark red) and mild hypothermia (light red). The black and grey experimental data correspond to the physiological and mild hypothermia respectively. Glucose(A), Lactate(B), Asparagine(C), Ammonia(D), Glutamate(E) Glutamine(F) Alanine(G). |
