# Supplementary material for: Population balance modelling captures host cell protein dynamics in CHO cell cultures
Source: PLoS One. 2022 Mar 23;17(3):e0265886. doi: 10.1371/journal.pone.0265886 (PMC8959726; doi:10.1371/journal.pone.0265886)
Supplement: S1 Table — (DOCX) [file pone.0265886.s003.docx]

**Table S1**: Parameter values for Eq. (37).

| Parameter | Average cell volume function | SD of the cell volume function |
| --- | --- | --- |
| $\boldsymbol{A}_{\boldsymbol{1}}$ | 989 | 466 |
| $\boldsymbol{A}_{\boldsymbol{2}}$ | 3081 | 1028 |
| $\boldsymbol{x}_{\boldsymbol{0}}$ | 418 | 405 |
| $\boldsymbol{p}$ | 39 | 21 |
