# Supplementary material for: Population balance modelling captures host cell protein dynamics in CHO cell cultures
Source: PLoS One. 2022 Mar 23;17(3):e0265886. doi: 10.1371/journal.pone.0265886 (PMC8959726; doi:10.1371/journal.pone.0265886)
Supplement: S2 Table — (DOCX) [file pone.0265886.s004.docx]

**Table S2**: Parameter values with their 95% confidence interval and units.

| 37^o^C | | 95% CI | 32^o^C | | 95% CI | Unit |
| --- | --- | --- | --- | --- | --- | --- |
| $\boldsymbol{\mu}_{\boldsymbol{max}}$ | $0.041$ | $2.530\times{10}^{-5}$ | $\boldsymbol{\mu}_{\boldsymbol{max}}$ | $0.014$ | $1.82\times{10}^{-3}$ | $h^{-1}$ |
| $\boldsymbol{K}_{\boldsymbol{asn}}$ | $0.33$ | $0.0151$ | $\boldsymbol{k}_{\boldsymbol{asn}}$ | $0.33$ | $0.0151$ | $mM$ |
| $\boldsymbol{K}_{\boldsymbol{amm}}$ | $5.9$ | $0.5$ | $\boldsymbol{k}_{\boldsymbol{amm}}$ | $5.9$ | $0.44$ | $mM$ |
| $\boldsymbol{k}_{\boldsymbol{d max}}$ | $0.013$ | $4.42\times{10}^{-5}$ | $\boldsymbol{k}_{\boldsymbol{d max}}$ | $0.011$ | $6.87\times{10}^{-4}$ | $h^{-1}$ |
| $\boldsymbol{k}_{\boldsymbol{d}}$ | $0.0088$ | $1.29\times{10}^{-4}$ | $\boldsymbol{k}_{\boldsymbol{d}}$ | $0.0036$ | $2.6\times{10}^{-4}$ | $h^{-1}$ |
| $\boldsymbol{k}_{\boldsymbol{l}}$ | $0.0084$ | $4.390\times{10}^{-5}$ | $\boldsymbol{k}_{\boldsymbol{l}}$ | $0.0048$ | $2.3\times{10}^{-4}$ | $h^{-1}$ |
| $\boldsymbol{Y}_{\boldsymbol{asn}}$ | $7.8\times{10}^{-10}$ | $5.18\times{10}^{-13}$ | $\boldsymbol{Y}_{\boldsymbol{asn}}$ | $1.9\times{10}^{-9}$ | $1.35\times{10}^{-10}$ | $mmol$ ${cell}^{-1}$ |
| $\boldsymbol{Y}_{\boldsymbol{amm/asn}}$ | $0.55$ | $0.00678$ | $\boldsymbol{Y}_{\boldsymbol{amm/asn}}$ | $1.8$ | $0.181$ | $mmol{mmol}^{-1}$ |
| $\boldsymbol{Y}_{\boldsymbol{amm}}$ | $5.4\times{10}^{-12}$ | $1.4\times{10}^{-13}$ | $\boldsymbol{Y}_{\boldsymbol{amm}}$ | $2.6\times{10}^{-11}$ | $5.3\times{10}^{-12}$ | $mmol$ ${cell}^{-1} h^{-1}$ |
| $\boldsymbol{k}_{\boldsymbol{amm}}$ | 3.5 | 0.22 | $\boldsymbol{k}_{\boldsymbol{amm}}$ | $3.5$ | $0.071$ | $mM$ |
| $\boldsymbol{Y}_{\boldsymbol{glc}}$ | $2.6\times{10}^{-9}$ | $3.55\times{10}^{-10}$ | $\boldsymbol{Y}_{\boldsymbol{glc}}$ | $2.2\times{10}^{-9}$ | $1.8\times{10}^{-12}$ | $mmol$ ${cell}^{-1}$ |
| $\boldsymbol{m}_{\boldsymbol{glc}}$ | $1.6\times{10}^{-11}$ | $5.5\times{10}^{-12}$ | $\boldsymbol{m}_{\boldsymbol{glc}}$ | $1.6\times{10}^{-11}$ | $3.13\times{10}^{-13}$ | $mmol {cell}^{-1}$ $h^{-1}$ |
| $\boldsymbol{m}_{\boldsymbol{mAbs}}$ | $8.5\times{10}^{-13}$ | $5.854\times{10}^{-15}$ | $\boldsymbol{m}_{\boldsymbol{mAbs}}$ | $8.5\times{10}^{-13}$ | $3.61\times{10}^{-15}$ | $g$ ${cell}^{-1} h^{-1}$ |
| $\boldsymbol{Y}_{\boldsymbol{lac/glc}}$ | $1.4$ | 0.318 | $\boldsymbol{Y}_{\boldsymbol{lac/glc}}$ | $1.2$ | 0.02 | $mmol{mmol}^{-1}$ |
| $\boldsymbol{m}_{\boldsymbol{lac}}$ | $5.6\times{10}^{-11}$ | $1.49\times{10}^{-11}$ | $\boldsymbol{m}_{\boldsymbol{lac}}$ | $4.5\times{10}^{-11}$ | $2.48\times{10}^{-12}$ | $mmol {cell}^{-1} h^{-1}$ |
| $\boldsymbol{Y}_{\boldsymbol{ala}}$ | $3\times{10}^{-10}$ | $8\times{10}^{-10}$ | $\boldsymbol{Y}_{\boldsymbol{ala}}$ | $7.7{\times10}^{-10}$ | $2.5\times{10}^{-10}$ | $mmol$ ${cell}^{-1}$ |
| $\boldsymbol{Y}_{\boldsymbol{gln}}$ | $1.8\times{10}^{-10}$ | $1.92\times{10}^{-12}$ | $\boldsymbol{Y}_{\boldsymbol{gln}}$ | $5.9\times{10}^{-10}$ | $9.2\times{10}^{-11}$ | $mmol {cell}^{-1}$ |
| $\boldsymbol{Y}_{\boldsymbol{glu}}$ | $1.1\times{10}^{-10}$ | $3.76\times{10}^{-12}$ | $\boldsymbol{Y}_{\boldsymbol{glu}}$ | $2.4\times{10}^{-10}$ | $3.4\times{10}^{-11}$ | $mmol$ ${cell}^{-1}$ |
| $\boldsymbol{q}_{\boldsymbol{HCP}}$ | $2.4\times{10}^{-13}$ | $1.1\times{10}^{-14}$ | $\boldsymbol{q}_{\boldsymbol{HCPs}}$ | $3.4{\times10}^{-14}$ | $3.3\times{10}^{-14}$ | $g {cell}^{-1} h^{-1}$ |
| $\boldsymbol{q}_{\boldsymbol{HCP}\left( \boldsymbol{2} \right)}$ | $7.5\times{10}^{-13}$ | $1.3\times{10}^{-13}$ | $\boldsymbol{q}_{\boldsymbol{HCPs}\left( \boldsymbol{2} \right)}$ | $1.5\times{10}^{-11}$ | $3.1\times{10}^{-12}$ | $g {cell}^{-1} h^{-1}$ |
